# Beyond Flat: Undulated Perovskite Solar Cells on Microscale Si Pyramids by Solution Processing

**DOI:** 10.1021/acsenergylett.5c00221

**Published:** 2025-02-27

**Authors:** Deniz Turkay, Kerem Artuk, Mostafa Othman, Florent Sahli, Lisa Champault, Christophe Allebé, Aïcha Hessler-Wyser, Quentin Jeangros, Christophe Ballif, Christian M. Wolff

**Affiliations:** † École Polytechnique Fédérale de Lausanne (EPFL), Institute of Electrical and Micro Engineering (IEM), Photovoltaics and Thin-Film Electronics Laboratory (PV-lab), Rue de la Maladière 71b, 2000 Neuchâtel, Switzerland; ‡ CSEM, Sustainable Energy Center, Rue Jaquet-Droz 1, 2002 Neuchâtel, Switzerland

## Abstract

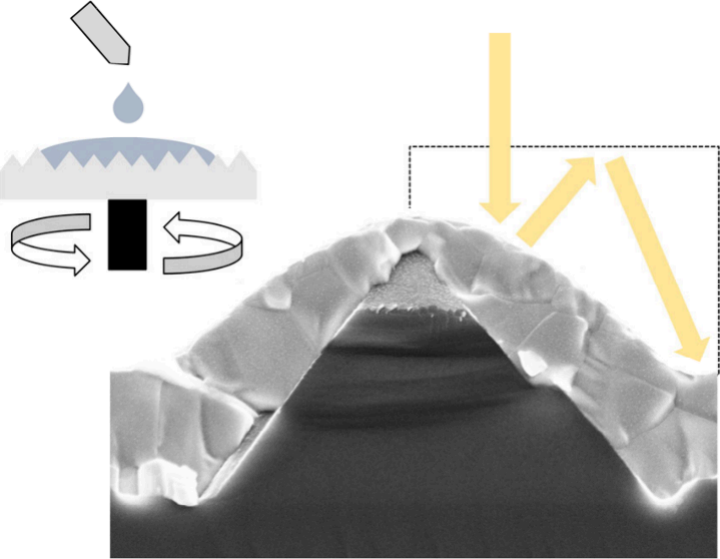

Microscale pyramids of silicon solar cells are often considered incompatible with solution-processed perovskite films. Thus, solution processing has mainly been used with submicron pyramids that are buried under thick perovskite films with flattened front surfaces. Yet, while this modification simplifies the fabrication process, it compromises optical performance compared to conformal perovskite films (e.g., obtained by vapor processing). Here, we show that protrusion-free perovskite films can be formed on random pyramids much higher than the film thickness by tailoring the film thickness to match the pyramids’ height profile, notably without modifying the pyramid facets. Accordingly, we demonstrate perovskite cells spin-coated on over 2 μm-high random pyramids with electronic performance comparable to those fabricated on flat substrates, and proof-of-concept perovskite-silicon tandem devices with efficiencies reaching 33%. Finally, we show that the undulated films with enhanced conformality enable optical performance superior to flat surfaces, especially upon encapsulation, most relevant to outdoor applications.

As perovskite-silicon (Si) tandem devices ramp up in power conversion efficiencies (PCEs) and approach industrialization, the ideal surface configuration for the Si bottom cell and the method for depositing the perovskite absorber on top continue to be subjects of scientific debate. For single junction Si solar cells, the random pyramids at the front surface are an industry standard due to the high optical performance that can be achieved via alkaline texturing. However, these textures typically have features that are locally far higher than the thicknesses of the commonly used perovskite films, which can lead to incomplete coverage of the pyramids and, consequently, shunting. As a result, two main fabrication approaches for combining perovskite films with Si pyramids have emerged over the years—(i) using Si textures smaller than the industry standard (e.g., with local heights less than about 1 μm) and burying the pyramids with a relatively thick perovskite layer that is roughly planarized on the front surface or (ii) employing vapor-based deposition techniques, optionally in combination with solution-based ones, to obtain near conformal films on the textures.^1−6^ The conformal coating theoretically yields the highest optical performance, yet typically comes at the cost of enhanced complexity in the fabrication procedure.^7,8^ A thick coating obtained by burying the pyramids, on the other hand, results in relatively enhanced front reflectance losses due to the near-planarized front surface. Pyramids with feature sizes smaller than the near-IR wavelengths might also result in reduced optoelectronic performance for the bottom cell, which can require asymmetric texturing of the front and rear sides of the wafers, increasing processing complexity.^9^ Compared to these main approaches, solution-processing on microscale pyramids and obtaining films with surface angles approaching those of vapor-deposited films remain relatively unexplored routes. A notable recent example in this category is that of Ying et al., introducing nanotextures on microscale pyramid facets by metal-assisted chemical etching while simultaneously reducing the surface angle of the pyramids below 40° to achieve near-conformal coverage of the perovskite films and tandem devices with PCEs reaching 30%.^10^ Another example is that of Farag et al., providing valuable insights on variables (e.g., of the solvent system, hole transport layer configuration) that affect crystallization and the recombination losses of thick perovskite films deposited on microscale textures, yet with relatively brief discussions on variables that are critical for pyramid coverage and conformality, and without demonstration of functioning devices.^11^ In this work, we use solution-processing to deposit perovskite films on microscale pyramid textures to obtain distinctively undulated front surfaces, notably without any modification to the Si surface or changes to the material composition of the perovskite cell.

To understand the working principles of such an architecture, accurate assessment of the dimensions of the pyramids that accommodate the perovskite films is crucial. In this regard, dedicated studies on the measurement and analysis of pyramid size distribution can be found in the literature on Si solar cells, primarily with the goal of modeling these structures more accurately (e.g., for computational purposes) or as a quality control check for the texturization step.^12−14^ For perovskite-Si tandem applications, however, there is no consensus on a quantitative description of pyramid dimensions that are critical for processing the perovskite films nor is there an associated definitive measurement methodology. Considering commonly used figures of merit, the maximum and average pyramid height for a single pyramid are the prominent ones. Maximum height is defined as the vertical distance from the tip to the lowest point at one of the four pyramid bases, while average height is the mean vertical distance from the tip to the base along all four facets or edges of the pyramid. (Figure 1a, b). As for their experimental measurement, two approaches stand out: (i) cross-sectional scanning electron microscopy (SEM) imaging that enables a two-dimensional analysis and (ii) atomic force microscopy or top-view SEM imaging enabling a three-dimensional analysis.^15,16^ While cross-sectional imaging can provide information on the maximum pyramid height of a wafer, the method can require a substantial number of images for reliable statistics and is more suited for a pass-fail type, semiqualitative analysis to determine whether a certain maximum height requirement is satisfied. The top-view images (e.g., Figure 1a), on the other hand, contain substantially more information within a single image and can provide a greater degree of statistical data, in particular when combined with a suited image processing algorithm.^14,17^ More importantly, these images contain crucial information inherently missing in the cross-sectional images—the dimensions of all four facets of a single pyramid. Our hypothesis is that even if some of the facets of a pyramid are particularly long, leading to tip-to-base heights locally much greater than the thickness of the perovskite film, the presence of a small facet on the same pyramid—together with a neighboring pyramid in direct contact—can still enable full coverage (i.e., without exposed pyramids) by promoting growth conditions and film thicknesses that are locally similar to those on a planar substrate, especially near the intersection of the two pyramids, allowing the formation of a film thick enough to cover the tips of the pyramids (Figure 2b). Accordingly, the minimum height difference between the tip and the base of a pyramid, and how this compares to the perovskite film thickness, should be more decisive for protrusion-free film formation rather than the maximum, or the average height. In other words, a recipe producing a perovskite film with a thickness comparable to the upper bound of the minimum pyramid height should be sufficient for full coverage of the pyramids.

**Figure 1:**
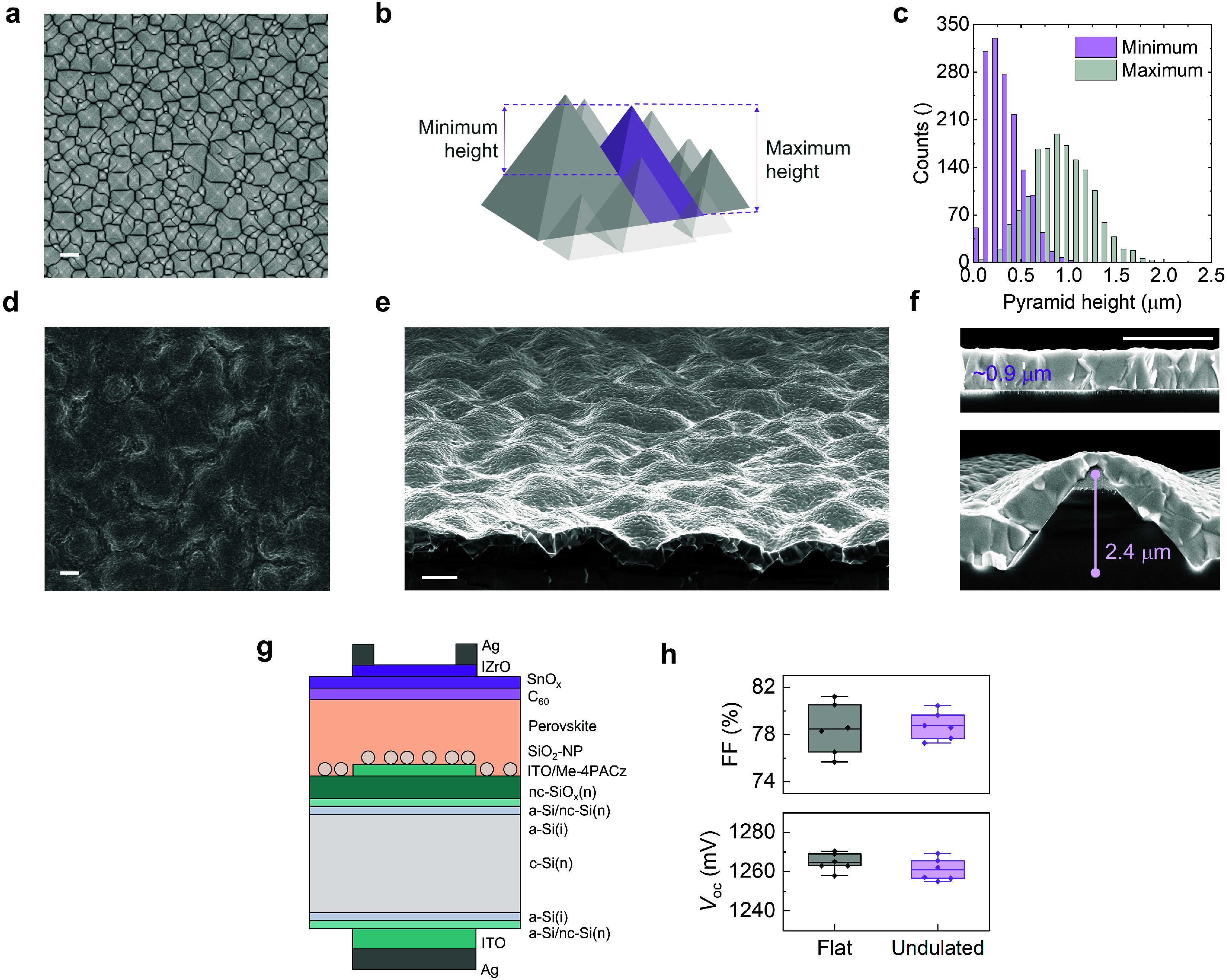
(a) Top view scanning electron microscope (SEM) image of a microscale pyramid textured Si wafer. (b) Tilted cross-section schematic of random pyramids depicting the minimum and maximum heights for a single pyramid. (c) Histogram of minimum and maximum pyramid heights extracted from 6 top-view images taken from random locations on the wafer with the same magnification. (d) Top view and (e) cross-section SEM images of perovskite-covered pyramids with a tilt of 10°. (f) Close-up cross-section SEM images of the perovskite film coated with the same recipe on flat and textured wafers. (g) Cross-section schematic of single-junction perovskite cells on ohmic Si substrates. (h) Fill factor and open-circuit voltage of 0.4 cm^2^ cells on the ohmic Si substrates. The scale bars in all SEM images are 2 μm in width.

**Figure 2:**
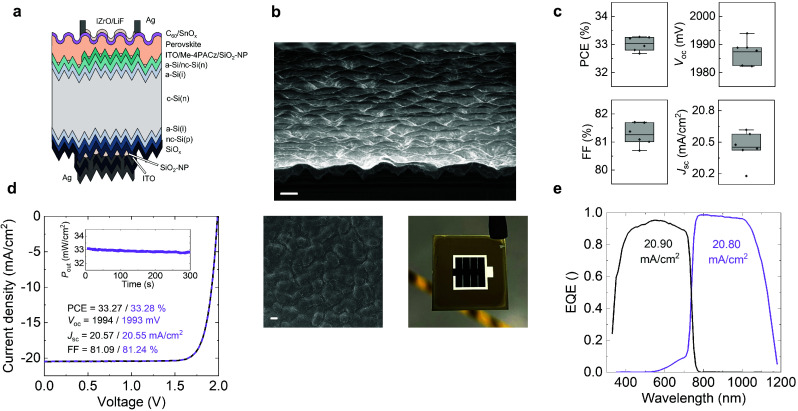
Cross-section (a) schematic and (b) tilted (10°) cross-section and top-view scanning electron microscope (SEM) images of the front side, and a photograph of a perovskite-Si tandem device. The scale bars are 2 μm in width. (c) Power-conversion efficiency (PCE), open-circuit voltage (*V*_oc_), fill factor (FF) and short-circuit current density (*J*_sc_) of the 1 cm^2^ tandem devices. (d) Current density–voltage characteristics and the power output (*P*_out_) measured by maximum-power-point tracking for the highest-performing device. The black curve is for the scan in the direction of *J*_sc_ to *V*_oc_, and the dashed purple curve is for *V*_oc_ to *J*_sc_. (e) Example external quantum efficiency (EQE) spectra of the subcells measured from the nonmetalized part of a tandem device.

To test our hypothesis, we use a microscale texture profile that is already used as a standard with our single-junction Si heterojunction devices (Figure 1a)^18,19^ and notably with our hybrid vapor–solution-processed perovskite-Si tandem devices.^4^ The height profiles of these pyramids were extracted by combining top-view SEM images with a human-supervised image processing algorithm, revealing minimum and maximum heights of less than 1 and 2.5 μm, respectively (Figure 1c, Figure S1). Next, we deposited the perovskite films on both flat and microscale-textured surfaces using a relatively simple one-step spin-coating recipe (i.e., 3000 rpm, 500 rpm/s, 40 s) (Figure 1d–f). By varying the perovskite ink concentration and thus the film thickness, we observed that the onset of protrusion-free film formation indeed occurs at thicknesses significantly smaller than the maximum pyramid height of the textured surface (Figure S2). Full coverage could already be achieved with film thicknesses of ∼0.9 μm, aligning more closely with the upper bound of the minimum pyramid height. Notably, the same correlation was also observed combining submicron textures with thinner films (Figure S3).

We note, however, that factors such as the spin-coating profile, ink composition and surface wettability can still play a role in protrusion-free coverage of pyramids with films that are thinner than the minimum pyramid height. For example, excessively high acceleration and speed during spin-coating can result in protrusions appearing when the film thickness falls slightly below the upper bound of the minimum height, demonstrating low tolerance for thickness variations around this threshold (Figures S3 and S4). In contrast, slowing down the spin profile particularly in the initial stages (e.g., with a multistep recipe) can enable full coverage even with extra-thin films or extra-large pyramids (e.g., maximum heights up to 4 μm) (Figures S5 and S6). Additionally, a complete modification of the pyramid facets (e.g., reinforcement by hydrophilic nanoparticles), can also improve the perovskite film coverage (Figure S7). Nonetheless, we confine the main discussions here to the relatively simple fabrication procedure to show that such advanced modifications are not a prerequisite for achieving full coverage and high optoelectronic performance with the microscale textures used in this study.

Having identified the conditions required to achieve protrusion-free films on microscale-textured surfaces, we then investigated the device performance with these films. For this, we used a Cs_0.05_(FA_0.9_MA_0.1_)_0.95_Pb(I_0.8_Br_0.2_)_3_ + 3% MAPbCl_3_ absorber in combination with piperazinium chloride (PCl) for top interface passivation, which enables outstanding device performances, with details that can be found elsewhere.^20,21^ In terms of the device architecture, we used single-junction perovskite cells on ohmic Si substrates (Figure 1g, Figure S8), which are particularly suited for assessing the performance of different perovskite cell configurations without the influence of a rectifying bottom cell. Our best-performing flat devices typically incorporate Me-4PACz as a hole transport layer (HTL) along with sparsely distributed SiO_2_ nanoparticles (SiO_2_–NPs) to enhance wettability and device efficiency—an approach that can also be transferred to textured structures (Figure S9).^22^ While the wettability of the perovskite ink on a textured surface coated with ITO and Me-4PACz is improved compared to a flat surface, the use of NPs further improve the wettability and the surface coverage of the 2.5 × 2.5 cm^2^ substrates (Figure S10). Notably, when deposited on pyramids covered with ITO/Me-4PACz, the NPs assemble at the bottom of the valleys, without sticking to the facets, and a microscale difference in perovskite film coverage (e.g., of the tips) is not observed due to their use (Figure S9). Achieving protrusion-free films and similarly performing devices is also possible without the use of NPs (see Figure 1, Figures S2, S5, S6, S11). Overall, the electronic performances achieved with the perovskite films on the microscale textures are similar to those coprocessed on flat surfaces with the same substrate configuration besides the surface morphology (Figure 1h). Moreover, these performances on textures substrates can be achieved repeatably (Figures S12 and S13).

We also fabricated two-junction perovskite-Si tandem devices (1 cm^2^) with this configuration to demonstrate the efficiency potential with functional bottom cells. In this case, the rear side is completed with a p–n junction, a SiO_2_–NP rear reflector and thick protective layers to enhance the average device performance and repeatability (Figure 2a,b).^22,23^ The index matching nc-SiO_*x*_(n) layer on the Si cell, which is crucial for flat devices, is omitted since the reflectance at the perovskite-Si interface is already suppressed to a large extent due to texturing. Overall, the proof-of-concept tandem devices yield an average FF, *V*_oc_, *J*_sc_ and PCE of about 81%, 1985 mV, 20.5 mA/cm^2^ and 33%, respectively (Figure 2c). Notably, there remains room for improvement in the *J*_sc_ of these devices which feature relatively thick electron transport layer stacks at the front side of both the perovskite and the Si cells (i.e., 15 nm-thick C_60_ and a 20 nm-thick SnO_*x*_, a 25 nm-thick nc-Si(n) layer) which have not been optimized for this specific configuration. Improvements in these regions are likely to further improve the PCEs that can be attained with these devices.^20^ In terms of stability, preliminary results show that these devices exhibit similar performance to flat devices during continuous operation, yet with shorter shelf lifetimes (i.e., 3% relative PCE loss in 2 months of storage) (Figure S14). Since all functional layers of the perovskite cell undergo structural changes compared to their configuration in a flat cell, multiple factors could be contributing to this behavior (e.g., incomplete coverage of the electron or hole transport layers on the undulated and pyramid-textured surfaces). Further investigation is crucial to identify these factors and implement targeted modifications to improve the devices in this regard.^24−26^

A subject of interest specific to these undulated films is how their optical performance compares to some of the well-known configurations. For this comparison, we spin-coated perovskite films on flat, submicron-textured and microscale-textured wafers using the same ink and spin recipe, resulting in the full-flat, buried and undulated configurations (Figure 3a). As a reference, we also fabricated near-conformal films on the microscale textures by vapor–solution processing. Next, we measured the reflectance spectra of the samples using a spectrometer with an integrating sphere and near-normal light incidence (7°). For bare perovskite films deposited on Si, the interference fringes observed in the infrared wavelength range are eliminated when textures are introduced (Figure 3b). However, in the ultraviolet to visible wavelength range, where front-surface reflectance dominates the optical response, the reflectance remains nearly identical for the flat, buried, and undulated configurations. In contrast, the hybrid films exhibit a significant broadband reduction in reflectance. Given the distinctively higher conformality visible in the microscope images of the undulated samples, the lack of a notable difference in their reflectance spectra compared to front-side flat configurations is curious at first glance. Fortunately, the theoretical work of Gota et al. analyzing the optics of nonconformal perovskite coatings provide key insights for these experimental observations.^7^

**Figure 3:**
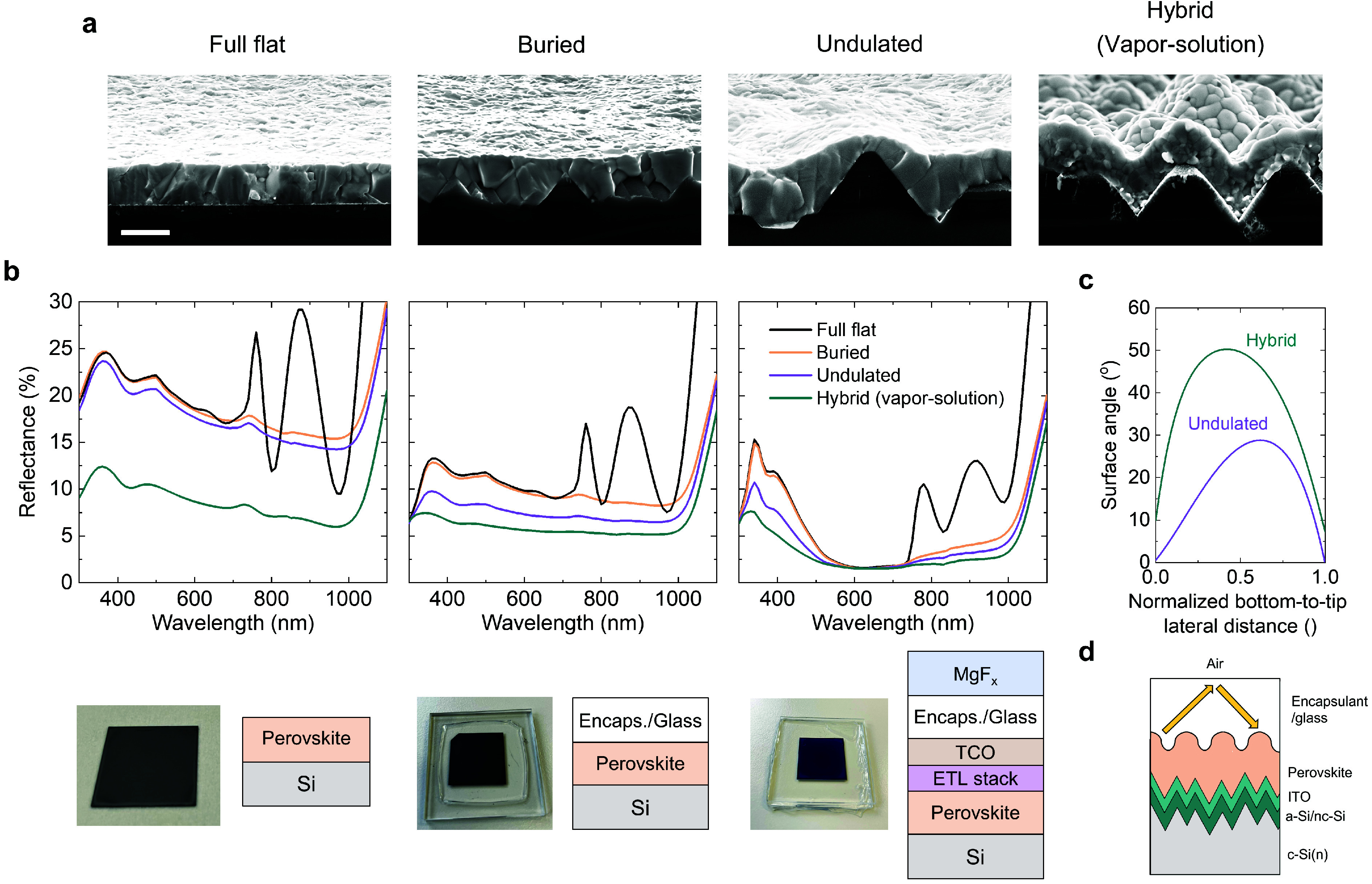
(a) Tilted cross-section scanning electron microscope (SEM) images of full-flat, buried, undulated and hybrid vapor–solution deposited, near conformal films. The scale bar is 1 μm in width. (b) Reflectance spectra of these configurations with the film stacks shown in the schematics underneath. (c) Example surface angle profile of the undulated and hybrid films for a single pyramid, calculated by processing the cross-section SEM images. (d) A cross-section schematic demonstrating the light undergoing total internal reflection upon being reflected from the textured surface.

The high optical performance of random upright Si pyramids is driven by the large surface angle (i.e., 54.7° theoretically), enabling normal incident reflected light to bounce from one pyramid to the adjacent one, striking the Si surface at least twice. A 45° angle ensures double-bounce for incident light, typically satisfied by the upright random pyramids. Below 45°, the probability of double-bounce rapidly decreases and becomes impossible below an angle of 30°. These critical angles also apply to perovskite-coated Si pyramids—the total reflectance off of a film with a front surface angle below 30° does not differ much from that with a full flat front side (i.e., 0°), although the light is scattered more for the former. In our case, the scenario is slightly more complicated since a single surface angle cannot be defined for the undulated perovskite films. Describing a local surface angle is more fitting for such an analysis (Figure 3c). To this end, we used cross-section SEM images to calculate the local surface angle (Figure S15). The results show that despite the distinct surface features, the surface angle is mostly limited to below 30° for the undulated films, although it can reach up to 40° in extreme cases (e.g., Figure 1f). This corresponds to a negligible benefit from the double-bounce effect, explaining the similar reflectance for the front-side flat and undulated films. For the hybrid samples, a large fraction of the surface angles indeed exceeds 45° (Figure 3c), though also with slight flattening at the tip and the bottom of pyramids.

While the as-deposited undulated films cannot meet the demanding surface angle requirements for the double-bounce effect, encapsulation of the samples presents an opportunity to benefit from the undulation in a real-world setting. Upon encapsulation, the possibility of total internal reflection (TIR) arises for light reflecting off the perovskite and obliquely striking the glass/air interface. Increased scattering from the undulated surface boosts the portion of light undergoing TIR, giving it a second chance to strike the perovskite film (Figure 3d). The lower limit for the surface angle to benefit from this effect is approximately 21°, corresponding to a relatively large portion of the undulated films.^7^ Accordingly, undulated samples demonstrate superior performance over front-side flat samples upon encapsulation, also with all the functional layers of a cell and an antireflective MgF_*x*_ film, and approach the performance of vapor–solution deposited films (Figure 3b). Finally, we emphasize that, while these measurements were conducted with near-normal light incidence in a laboratory setting, the angle of incidence varies with the motion of the sun during outdoor operation. In such a scenario, the energy yield is estimated to reach 99% of what can be achieved with fully conformal textures already for a surface angle of ∼24° and shows a more linear dependency on surface angle rather than a step-function-like behavior.^7^ Since this threshold is already met by the undulated films, their optical performance in real-world applications might not be far from ideal. However, further validation through outdoor experiments or advanced indoor measurements, such as scanning incidence angle reflectance measurements, is necessary to confirm these predictions.

In summary, we demonstrated here the possibility of obtaining high-performance undulated perovskite cells on microscale random upright pyramids with local heights much higher than the perovskite film thickness. Results show that complete film coverage can already be obtained with thicknesses roughly equal to the upper bound of the minimum pyramid height. Thickening the films beyond this threshold ensures tip coverage and reduces the sensitivity to deposition parameters, but at the expense of optical performance due to flattening of the front surface. In contrast, thinning the films below this threshold enables an increased degree of film conformality and additional optical gains that become particularly apparent upon encapsulation, yet also increases the sensitivity to deposition parameters and material configuration in obtaining protrusion-free films. By choosing a film thickness close to this threshold, a relatively simple fabrication sequence and an optical benefit from the undulated surface can be simultaneously achieved (Figure S16). We note, however, that the correlations observed here with spin-coating on random upright pyramids can differ if either the substrate morphology or the deposition conditions are changed, particularly if extra-thin films are used. For example, a reduced facet angle or smoothening of the sharp tips of the substrate can relax the requirements for full coverage.^8,10,24^ Scalable solution-based methods (e.g., blade, slot-die coating), which are relevant for commercialization, can exhibit differences in terms of the solvent system and crystallization dynamics and can include additional process variables that influence film coverage considerably (e.g., substrate heating).^2,25,26^ Uncovering the influence of these various parameters is vital for finding a generalized solution to the problem of achieving protrusion-free films with a high degree of conformality on textured surfaces. Finally, we emphasize the importance of understanding and improving prominent degradation mechanisms in these architectures, crucial for the industrial applicability of the findings.

## Supporting Information Available

Additional details on fabrication and characterization of the samples, (S1) example on pyramid height profiling, (S2) perovskite films with various ink concentrations coated on microscale pyramids, (S3) perovskite films coated on submicron textures, (S4) perovskite films coated on submicron textures with excessively high acceleration and spin speeds, (S5, S6, S7) examples on configurations with higher conformality, coatings on extra-large pyramids and pyramid facet modification, (S8) a photograph of single junction perovskite cells on an ohmic Si substrate, (S9) comparison of SiO_2_–NPs coated on flat and textured surfaces, (S10) contact angle measurements of flat and textured substrates coated with Me-4PACz and SiO_2_–NPs, (S11) performance parameters of single-junction perovskite cells with Me-4PACz/SiO_2_-NP vs 2PACz, (S12, S13) performance parameters for different device batches and their EQE spectra, (S14) shelf and operational stability of the perovskite-Si tandem devices, (S15) an example demonstrating the surface angle calculation, (S16) sketch of pyramid height, (S17) irradiance spectra of the solar simulator setup (PDF)
