# Supplementary material for: Beyond Flat: Undulated Perovskite Solar Cells on Microscale Si Pyramids by Solution Processing
Source: ACS Energy Lett. 2025 Feb 27;10(3):1397–403. doi: 10.1021/acsenergylett.5c00221 (PMC11915383; doi:10.1021/acsenergylett.5c00221)
Supplement: Supplementary file 1 — nz5c00221_si_001.pdf [file nz5c00221_si_001.pdf]

Supporting information

Beyond flat: Undulated perovskite solar cells on microscale Si pyramids by solution processing

Deniz Turkay[1], Kerem Artuk[1], Mostafa Othman[1], Florent Sahli[2], Lisa Champault[2], Christophe Allebé[2], Aïcha Hessler-Wyser[1], Quentin Jeangros[2], Christophe Ballif[1,2], Christian M. Wolff[1,$]*

1. École Polytechnique Fédérale de Lausanne (EPFL), Institute of Electrical and Micro Engineering (IEM), Photovoltaics and Thin-Film Electronics Laboratory (PV-lab), Rue de la Maladière 71b, 2000 Neuchâtel, Switzerland

2. CSEM, Sustainable Energy Center, Rue Jaquet-Droz 1, 2002 Neuchâtel, Switzerland

AUTHOR INFORMATION

Corresponding Author

* correspondence to: deniz.turkay@epfl.ch

$ correspondence to: christian.wolff@epfl.ch

Methods

Fabrication of Si bottom cells: The ohmic and functional (i.e., with a p-n junction) Si bottom cells were fabricated using 190 and 280 μm-thick, shiny-etched and mirror-polished monocrystalline Si wafers, respectively. For the textured samples, a random pyramid textured was formed using a potassium hydroxide solution, followed by wet chemical cleaning of the wafers. The height profiles of the textured wafers were measured by SEM to ensure consistency in pyramid dimensions. Prior to the PECVD processes, the wafers were dipped in a hydrofluoric acid solution to remove the thin chemical oxide at the surface. All hydrogenated amorphous and nanocrystalline Si layers were deposited by a plasma enhanced chemical vapor deposition (PECVD) system, with a plasma frequency of 13.56 MHz. On the front side, hydrogenated intrinsic and phosphorus-doped amorphous Si layers, [a-Si:H(i) and a-Si:H(n)], and the nanocrystalline phosphorus doped Si [nc-Si:H(n)] were deposited at 200 ºC. An additional 100 nm-thick nc-SiOx:H(n) layer was also included with both the front-side flat and textured ohmic substrates, and front-side flat tandems. On the rear side, the a-Si:H(i) deposition at 200 ºC was followed by the depositions of ultra-thin SiO_x and nc-Si:H(p) layers at 175 ºC for the functional cells. The rear side of the ohmic substrates were completed with an a-Si:H(i)/a-Si:H(n)/nc-Si:H(n) stack instead. The rear side of the ohmic Si cells were completed with a 80 nm-thick ITO and a 80 nm thick Ag layer. For the functional cells, first a 40 nm-thick ITO was deposited through a 1.1 × 1.1 cm^2 shadow mask on the rear side, then a 1 wt% SiO_2-NP dispersion was spin coated multiple times to fill the pyramid valleys to leave about 15% of the pyramid surface exposed for contacting with a 150 nm-thick sputtered Ag layer. The cells then received a full-area, 500 nm-thick SiO_x by PECVD, and an additional 150 nm-thick Ag to enhance their durability during perovskite processing. The front side of all cells were completed with a 20 nm-thick ITO sputtered through shadow masks with openings of 1.1 × 1.1 cm^2. The 4" wafers were then laser cut into 2.5 × 2.5 cm^2 substrates and annealed at 210 °C for 30 min to have them ready for processing perovskite cells on top.

Fabrication of perovskite films for coating tests, cells and perovskite-Si tandem cells: A triple-cation perovskite absorber $Cs_{0.05}FA_{0.90}MA_{0.10})_{0.95}Pb(I_{0.80}Br_{0.20})_3$, optionally with the addition of ~3% $MAPbCl_3$, was used in the study. While the precursor ink concentration (1.9 M) was kept the same for textured and flat samples for the coating tests on the correlation of film thicknesses and pyramid heights, a lower concentration (1.7M, our standard for flat surfaces) was used for flat devices to ensure higher film quality over the relatively large device areas. The precursor solutions of the triple-cation perovskite (TCP, $Cs_{0.05}FA_{0.90}MA_{0.10})_{0.95}Pb(I_{0.80}Br_{0.20})_3$) absorbers were prepared using four stock solutions of (a) 1.73 M (1.55) FAI (Dyenamo, >99.99%) and 1.9M (1.7)M PbI_2 (TCI, >99.99%) in DMF:DMSO = 4:1, (b) 0.866 (0.775) M FABr (Dyenamo, >99.99%) and 0.866 (0.775)M MABr (Dyenamo, >99.99%) and 1.9 (1.7)M $PbBr_2$ (TCI, >99.99%) in DMF:DMSO = 4:1, (c) 1.5M CsI (Alfa Aesar, >99.9%) in DMSO. Prior to the depositions, the solutions were mixed with a volume ratio of a:b:c = 80:20:4 respectively in order to obtain the TCP precursor. For the triple halide absorbers that also include an additional ~3% $MAPbCl_3$, 3 mg/mL MACl (TCI, >99.99%) and 12.5 mg/mL $PbCl_2$ (Dyenamo, >99.99%) were also added in the precursor solution at this stage. The Me-4PACz (TCI) were dissolved in ethanol (1 mg/mL). For the SiO_2-NP (~20 nm diameter) dispersion, synthetic amorphous colloidal silica was used. A 1 wt% stock supension was prepared in ethanol and further diluted by ethanol to obtain a concentration of 0.1 wt%. For interface passivation, 0.1 mg/mL piperazinium chloride (Dyenamo,>98%) was dissolved in IPA.

The Si cells first received a UV-ozone treatment for 30 min. The samples were then transferred to a N_2-filled glovebox, and Me-4PACz was spin-coated with a two-step recipe – first 75 μL of solution was dropped on the sample and rested for 10 sec, next the sample was spinned for 30 sec and 15 sec into the recipe 75 μL of SAM was again dropped on the sample. The sample were then dried on a hotplate at 100 ºC for 10 min. The SiO_2-NP was coated statically by spin coating at 2000 rpm with an acceleration of 500 rpm/s and drying on a hotplate at 100 ºC for 10 min. The perovskite precursor ink (100 μL) was dropped on the substrates right before spinning with a single-step recipe having an acceleration of 500 rpm/s, and peak rotation speed of 3000 rpm, lasting a total of 40 s. Anisole (300 μL) was dropped on the substrate when 10 s was remaining. The samples were then annealed on a hotplate at 100 ºC for 15 min. During the

processes, the glovebox temperature was 25 °C. To prevent excessive air flow affecting the perovskite film formation and drying, the air-conditioner was kept turned off during spin coating, and was only turned-on in between processing two samples. To control the ambient and prevent the build-up of solvent vapors, the foil in the spin coater was removed, sealed and renewed after each perovskite coating. The glovebox was continuously flushed during the depositions. After the perovkite depositions, PCl was spin coated dynamically on the samples at 6000 rpm for 30s, and they were annealed on the hotplate for 10 min. For the single-junction and tandem devices, a 20 and 15 nm-thick C_{60} layer (Creaphys, >99.9% - sublimed) was deposited by thermal evaporation, respectively. A 20 nm-thick SnO_x layer was deposited by atomic layer deposition at a temperature of 100 ºC. A 35 nm-thick IZrO was deposited as a transparent conductive oxide by radio-frequency sputtering through a shadow mask. Ag was thermally evaporated on the cells with a thickness of 400 and 800 nm for the single-junction and tandem devices, respectively, using shadow masks to produce the grids that are specific to the 0.4 and 1 cm^2 designs. For the perovskite-Si tandem devices, an additional 100 nm-thick LiF layer was thermally evaporated on the active area as an anti-reflection coating.

For the coating tests shown in **Fig. 1, S2, S5, S6**, which does not involve cell fabrication, no self-assembled monolayer or SiO_2-NPs were used on top of the ITO unless specified otherwise.

For the encapsulation of the optical test samples, a polyolefin film in combination with 2 mm-thick glass were used, and the lamination was performed at a temperature of 135 ºC.

Current-voltage measurements of the solar cells: The current-voltage measurements under simulated AM 1.5G illumination were conducted using a Wacom solar simulator (AAA) equipped with Xe and Ha lamps (**Fig. S16**). The intensities of the lamps were adjusted to match the subcell J_{sc}'s extracted from the EQE measurements, downscaled by 0.985 to account for front grid shadowing. A forward (i.e., near short circuit to open circuit) and reverse (i.e., near short circuit to open circuit) scan was conducted for each device. The terminal voltage was varied between -0.1 V and 1.3 (2.05) V for single-junction (tandem) devices at a scan speed of 200 mV/s. A temperature-controlled (25 ºC) brass chuck was used for contacting the rear side. The measurements were conducted with a Kelvin needle probe at the top side and with an isolated pin integrated in the brass chuck at the rear side, with a 4-wire configuration on each side. A rubber shadow mask was used to illuminate the active area on the Si substrates.

EQE measurements: Measurements were conducted with a custom-built spectral response setup where the samples were irradiated with chopped monochromatic light at a frequency of 217 Hz, with a spot size of ~1 mm^2, and the response was measured with a lock-in amplifier. For tandem cells, filtered light biases were applied to ensure that the subcell being measured was the current limiting one. A voltage bias between -0.5 to 0.5 V were applied during the measurement of each subcell.

SEM measurements: Images were acquired with acceleration voltages ranging from 1 to 5 kV using an in-lens detector (Zeiss GeminiSEM 450).

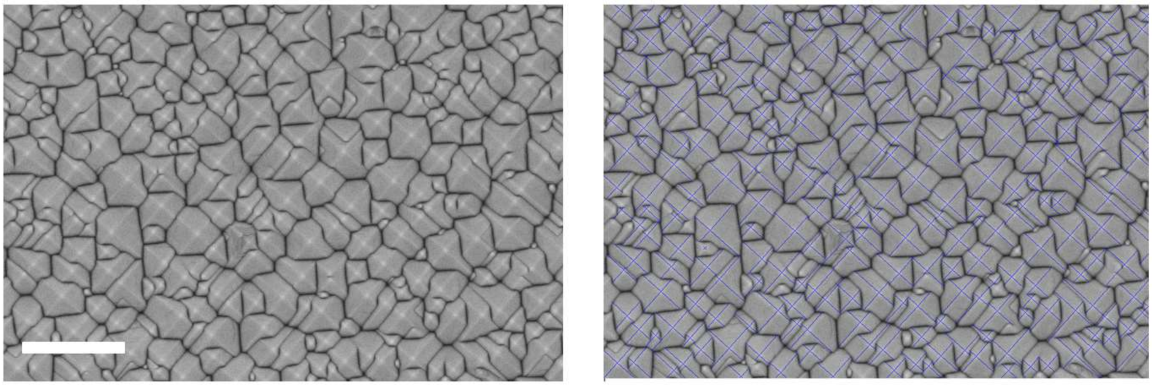

Fig. S1. An example of raw (left) and (right) processed top view SEM images of microscale pyramids. The blue lines show the detected edges for each pyramid. The tip-to-base distance (i.e. the height) corresponding to each edge was calculated assuming an edge angle of 45° (which should not be confused with the facet angle of 54.7°), and accordingly multiplying the extracted edge length with sin(45°). To enhance statistical reliability, images were taken from 6 randomly chosen locations on the wafer. The scale bar is 5 μm.

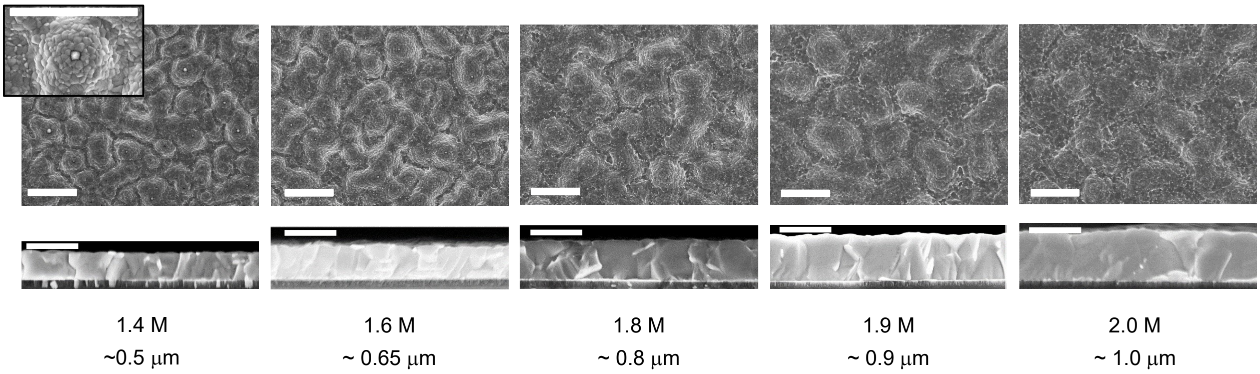

Fig. S2. Top-view and cross-sectional scanning electron microscope (SEM) images of pyramid textured (top row) and planar (bottom row) Si coated with perovskite ink precursors with varying concentrations, using the same spin recipe (3000 rpm, 500 rpm/s, 40 s). The scale bar is 5 μm in the top row, and 1 μm in the bottom row. The inset on the left shows a close-up image of a pyramid tip sticking out of the perovskite film.

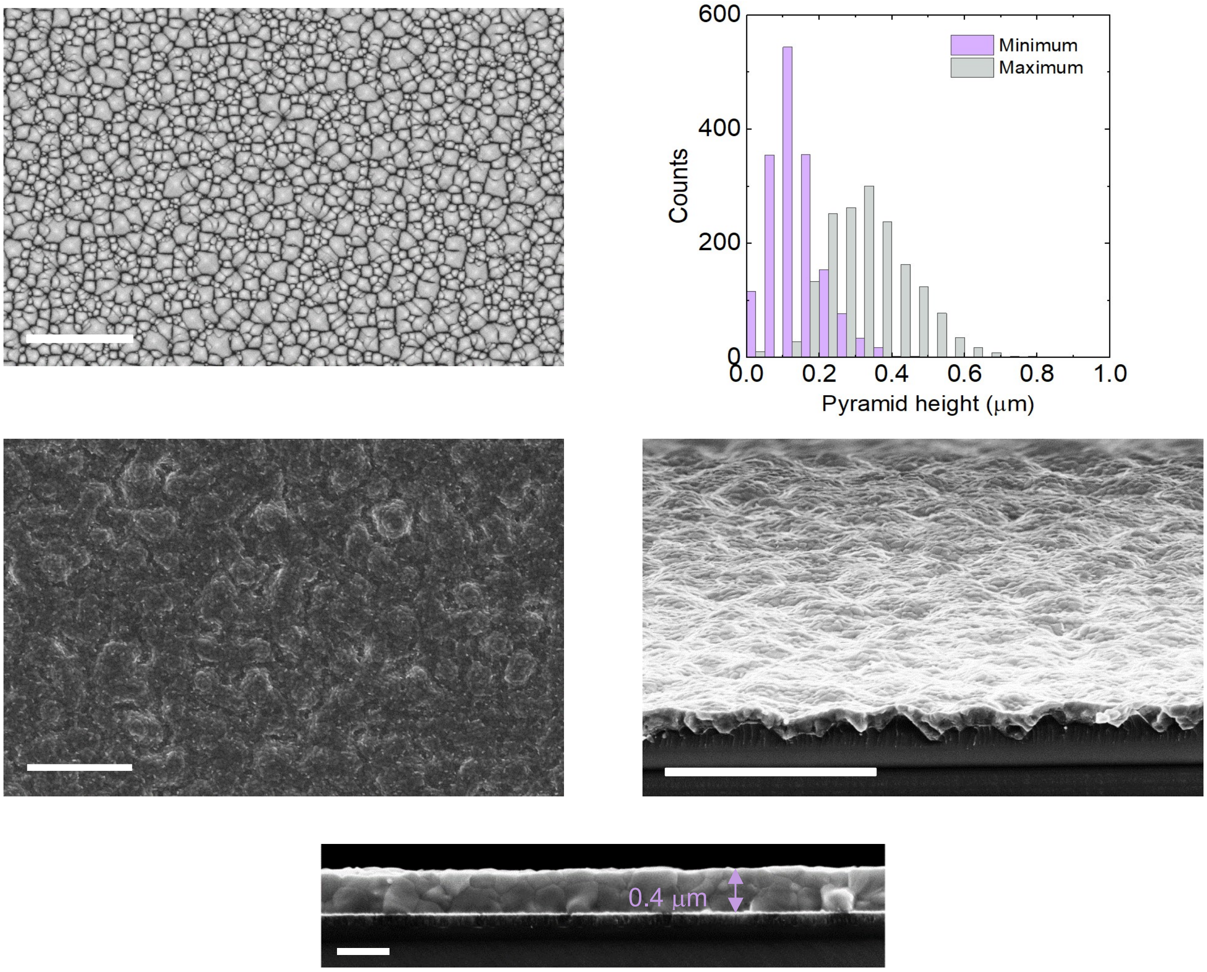

Fig. S3. (Top row) Top view scanning electron microscope (SEM) image of submicron textures and height distribution extracted from the top view SEM images of the textures. Scale bar is 5 μm. To reliably extract the height profile, images with double the magnification was used. **(Middle row)** Top view and cross section (with a tilt of 10°) SEM images of the textures coated with a perovskite film. Scale bars are 5 μm. **(Bottom row)** Cross-section image of the perovskite film when coated with the same recipe on a flat surface. Scale bar is 500 nm.

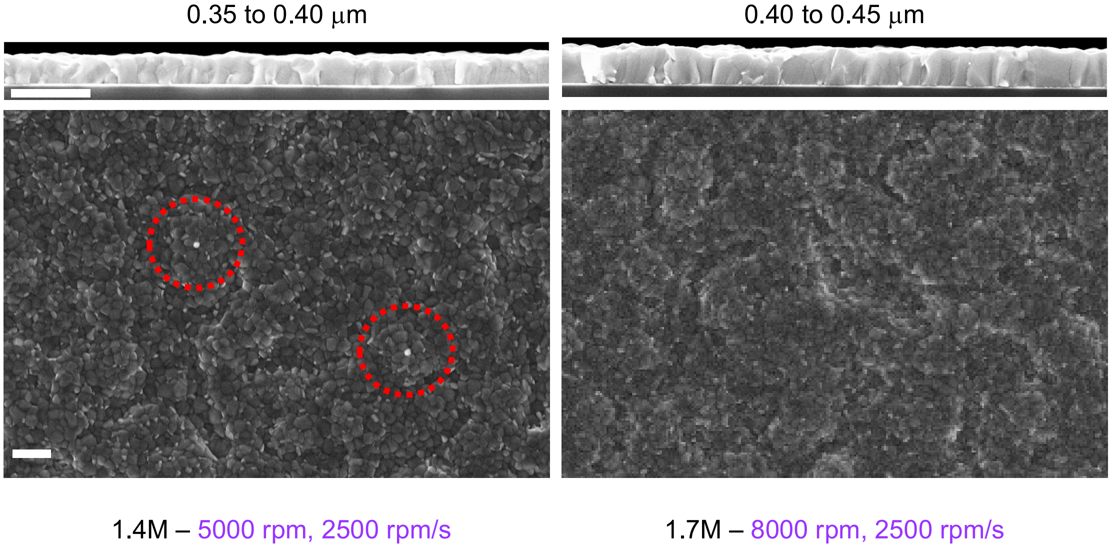

Fig. S4. (Top row) Cross-section and **(bottom row)** top-view scanning electron microscope (SEM) images of submicron textures shown in Fig. S3 coated at high acceleration and spin speeds, and with the precursor ink concentrations as specified below the images. Scale bars are 1 μm (same for the top and bottom rows).

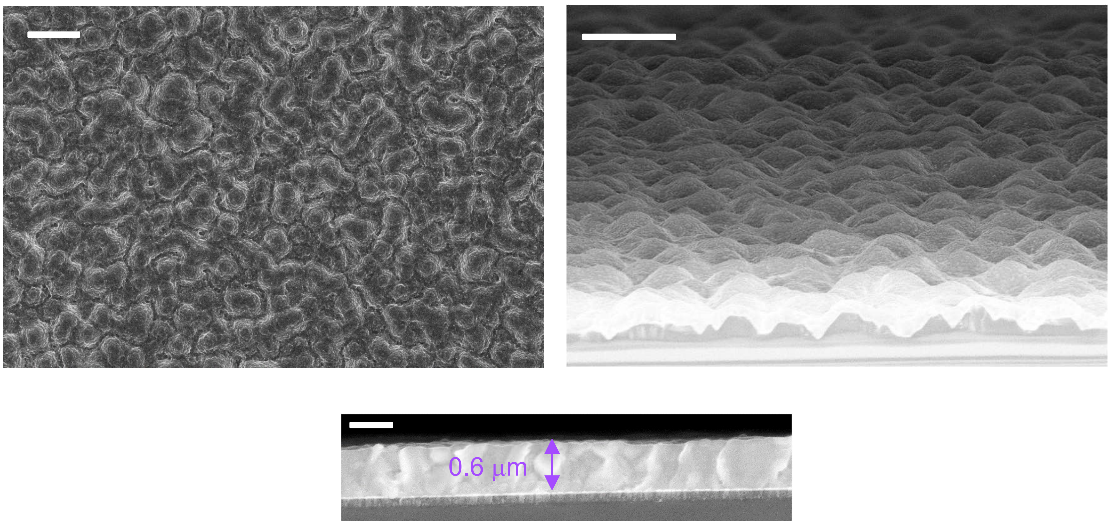

Fig. S5. A two-step spin coating recipe (1500 rpm, 500 rpm/s, 30s + 2500 rpm, 2000 rpm/s, 30s) combined with a low concentration perovskite solution (1.3 M) to enhance the level of surface curvature while ensuring full coverage of the pyramids (top row) with the thin films (bottom row).

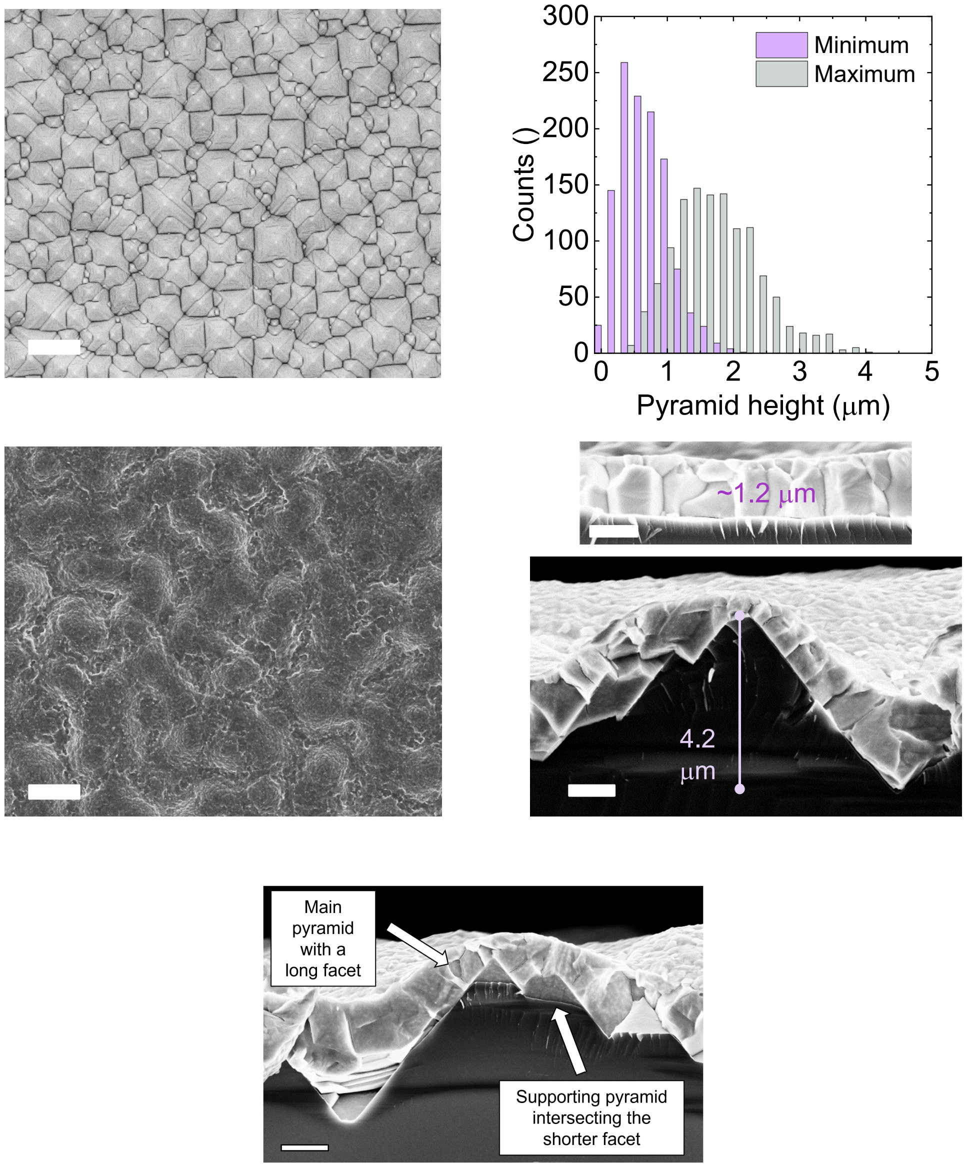

Fig. S6. (Top row) Top view scanning electron microscope (SEM) image of extra-large pyramid textures and height distribution extracted from the top view SEM images of the textures taken from 6 different locations on the sample. Scale bar is 5 μm. **(Middle row)** Top view (left) and cross section (right) SEM images of the samples coated with perovskite films, with a two-step recipe (2 M, 1500 rpm, 500 rpm/s, 30s + 2500 rpm, 2000 rpm/s, 30s). Scale bars are 1 μm. **(Bottom row)** Cross-section image of a perovskite film coated on the textures with annotations pointing out the support structure formed by neighboring pyramids enabling protrusion-free tip coverage. Scale bar is 1 μm.

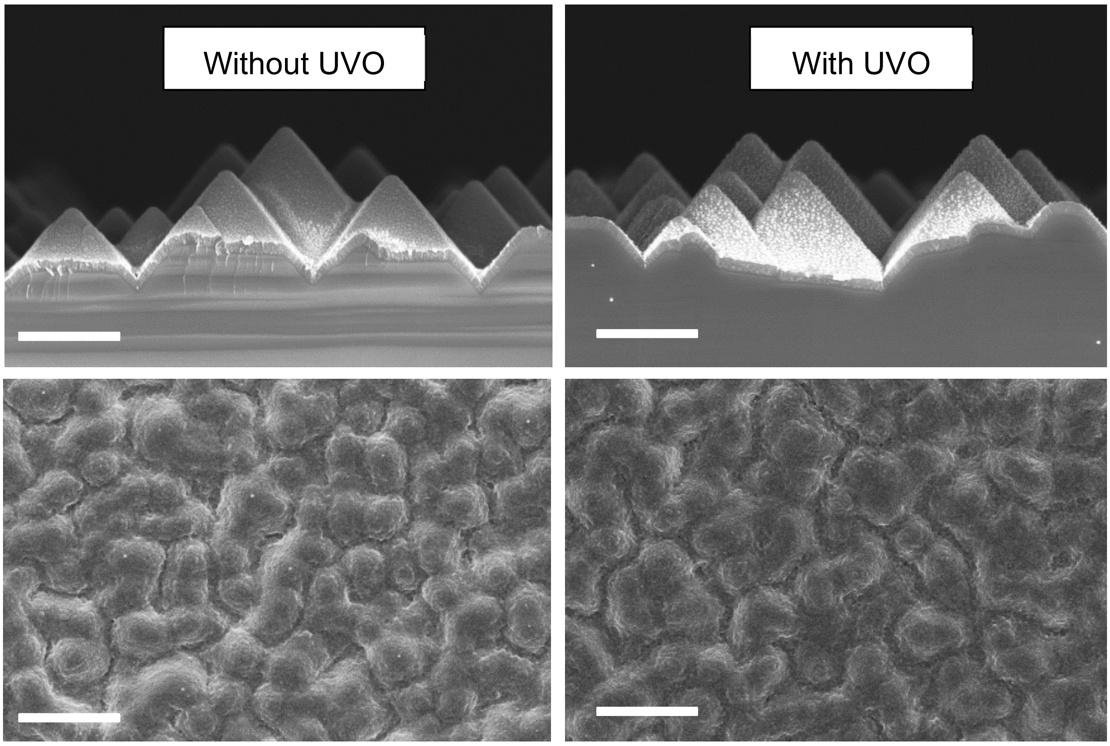

Fig. S7. **(Top row)** SiO_2-NPs coated on pyramid facets with and without the ultraviolet-ozone (UVO) activation prior to NP coating. The scale bar is 1 μm. **(Bottom row)** The top view SEM images of the perovskite films coated on the textures (0.9M, 500 rpm, 250 rpm/s, 60s + 2500 rpm, 2000 rpm/s, 30s). In the image on the left, the white spots on top of the curves are the pyramids slightly sticking out. The scale bar is 5 μm.

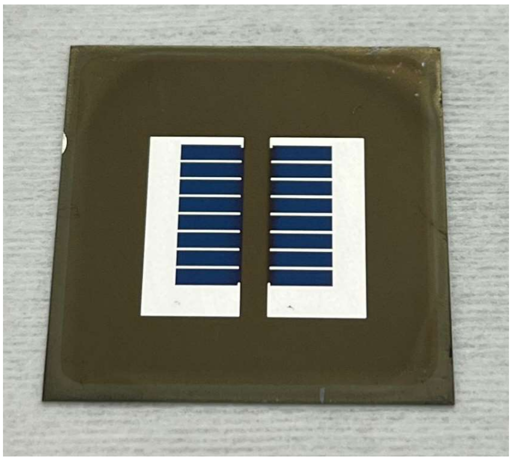

Fig. S8. Photograph of 0.4 cm^2 single-junction perovskite cells on the ohmic Si substrates.

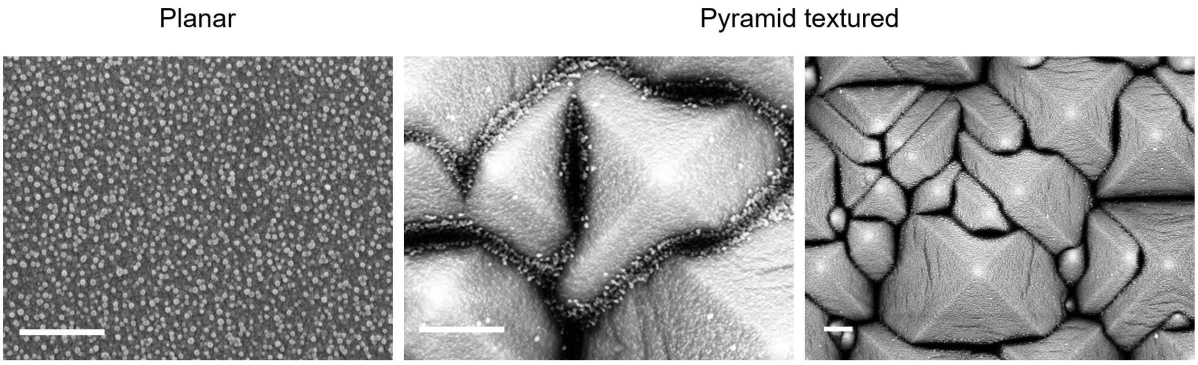

Fig. S9. Top view SEM images of SiO_2-NPs coated on flat (left) and pyramid textured (middle and right) surfaces covered with ITO/Me-4PACz. The scale bar is 500 nm.

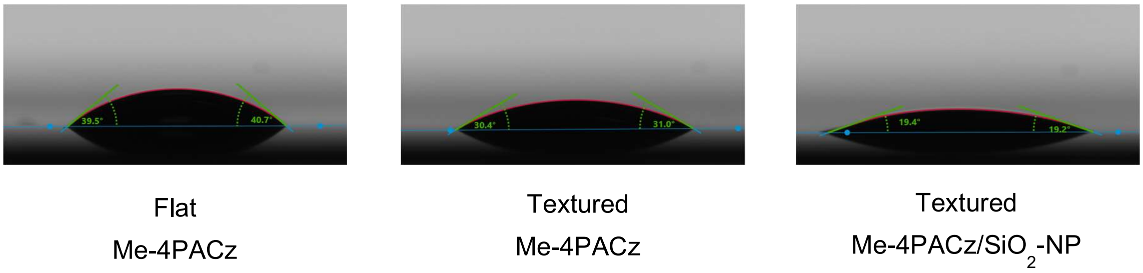

Fig. S10. Static contact angle measurements of perovskite ink droplets (1.9 M) dropped on flat and pyramid textured Si substrates coated with ITO/Me-4PACz and SiO_2-NPs (0.1 wt%).

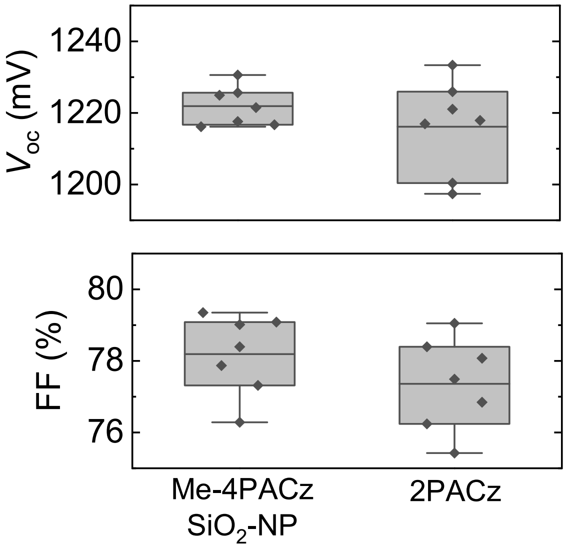

Fig. S11. Open-circuit voltage (V_{oc}) and fill factor (FF) of 1 cm^2 single-junction perovskite devices fabricated on microscale textured Si using two different hole transport layer configurations. Concentration of the used SiO_2-NPs is 0.1 wt%. In these experiments a double-halide absorber was used instead of the triple halide absorber.

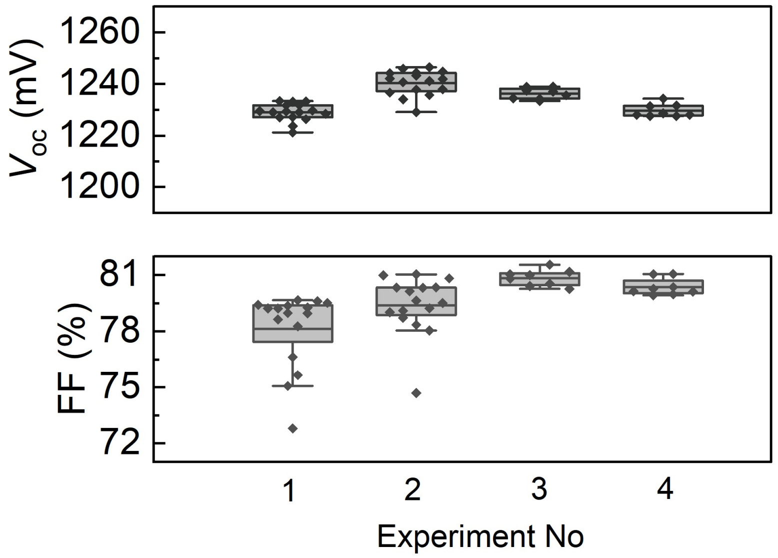

Fig. S12. Open-circuit voltage (V_{oc}) and fill factor (FF) of 0.4 cm^2 single-junction perovskite devices fabricated on microscale textured Si with ITO/Me-4PACz/SiO_2-NP as the HTL stack, consecutively in four experiments. In these experiments a double-halide absorber was used instead of the triple halide absorber.

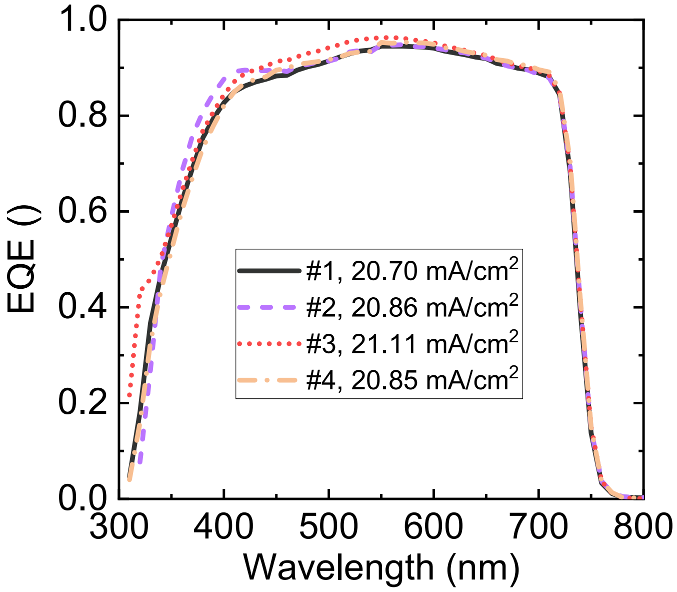

Fig. S13. External quantum efficiency (EQE) versus wavelength of single-junction perovskite devices on Si substrates fabricated over four batches with the performance parameters shown in Fig. S12, completed with LiF_x anti-reflection coating.

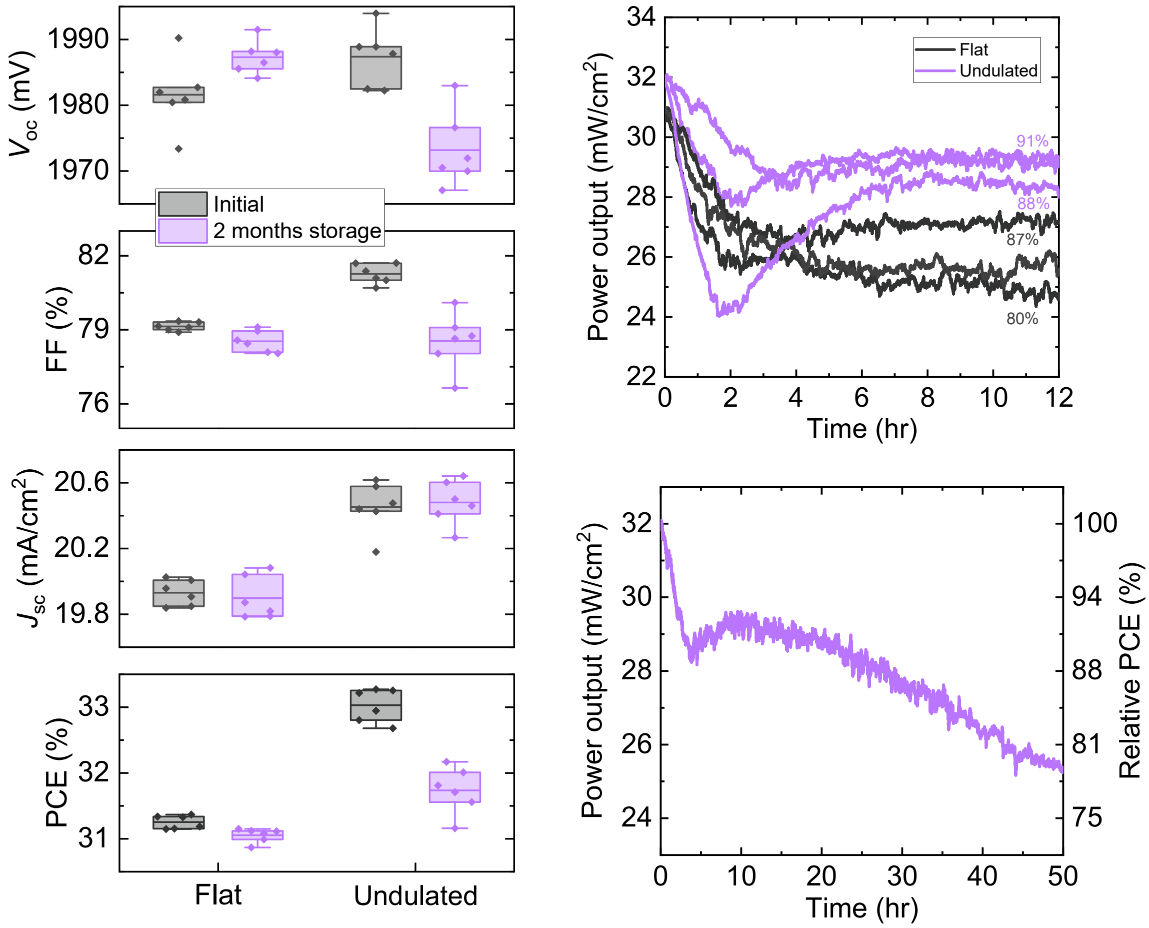

Fig. S14. **(Left)** Open-circuit voltage (V_{oc}), fill factor (FF), short-circuit current density (J_{sc}) and power conversion efficiency of 1 cm^2 perovskite-Si devices fabricated on front-side flat and microscale textured Si substrates, initially and after 2 months of storage in a N_2-filled glovebox. **(Right, top)** Power output of the unencapsulated and 2 month-aged devices measured by maximum power tracking for 12 hr under continuous 1-sun illumination. The relative power outputs (compared to the initial power output the cells) are specified in percentages in text. **(Right, bottom)** Power output of an undulated cell measured for 50 hr under the aforementioned conditions.

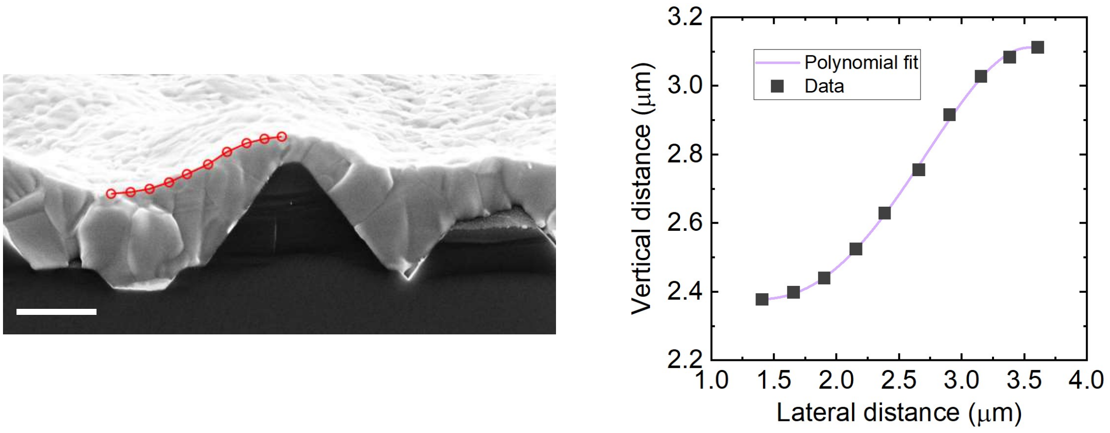

Fig. S15. Example of digitization and fitting of the surface lines of perovskite films from cross section SEM images. The surface angle was calculated from the slope of the curve that was fit by a polynomial that was used to smoothen the data.

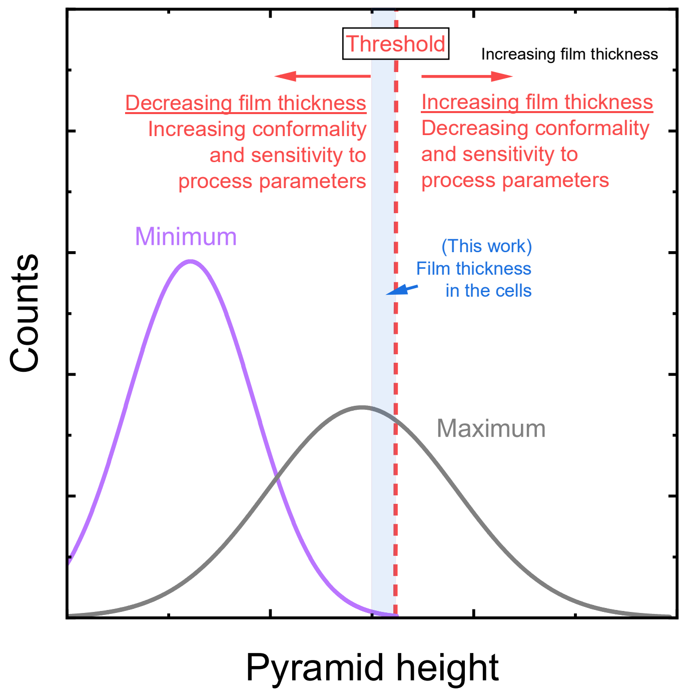

Fig. S16. Sketch of minimum and maximum pyramid height distributions and their correlation with perovskite film thickness, conformality and processing conditions.

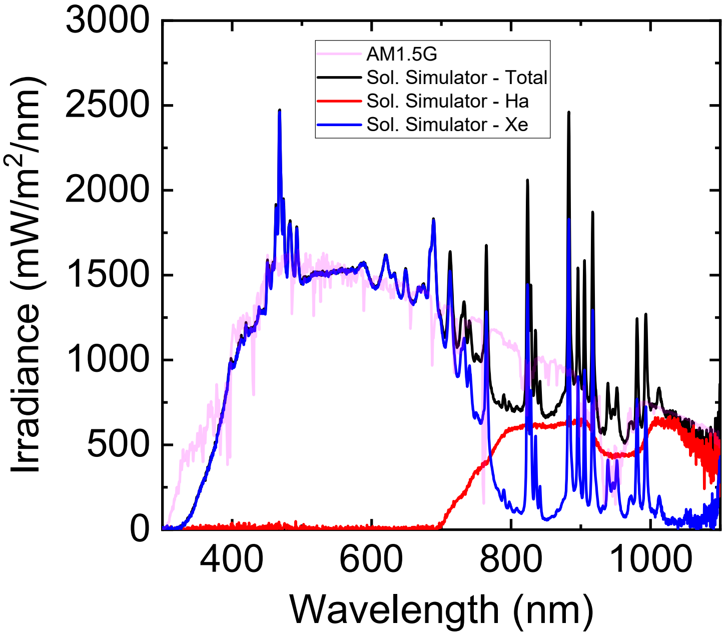

Fig. S17. Irradiance spectra of AM1.5G, and the two-source (Xe and Ha) solar simulator (WACOM) used for measuring the current-voltage characteristics of the solar cells.
